# Leveraging conversational technology to answer common COVID-19 questions

**DOI:** 10.1093/jamia/ocaa316

**Published:** 2021-02-01

**Authors:** Mollie McKillop, Brett R South, Anita Preininger, Mitch Mason, Gretchen Purcell Jackson

**Affiliations:** 1 IBM Watson Health, Cambridge, Massachusetts, USA; 2 Vanderbilt University Medical Center, Nashville, Tennessee, USA

**Keywords:** telemedicine, COVID-19, public health, pandemics, chatbots, conversational technologies

## Abstract

The rapidly evolving science about the Coronavirus Disease 2019 (COVID-19) pandemic created unprecedented health information needs and dramatic changes in policies globally. We describe a platform, Watson Assistant (WA), which has been used to develop conversational agents to deliver COVID-19 related information. We characterized the diverse use cases and implementations during the early pandemic and measured adoption through a number of users, messages sent, and conversational turns (ie, pairs of interactions between users and agents). Thirty-seven institutions in 9 countries deployed COVID-19 conversational agents with WA between March 30 and August 10, 2020, including 24 governmental agencies, 7 employers, 5 provider organizations, and 1 health plan. Over 6.8 million messages were delivered through the platform. The mean number of conversational turns per session ranged between 1.9 and 3.5. Our experience demonstrates that conversational technologies can be rapidly deployed for pandemic response and are adopted globally by a wide range of users.

## INTRODUCTION

A new coronavirus causing severe acute respiratory syndrome (SARS-CoV-2) has infected millions worldwide with coronavirus disease 2019 (COVID-19) and caused significant mortality. SARS-CoV-2 is a highly contagious pathogen, with widely variable clinical manifestations. In March 2020, the World Health Organization (WHO) classified COVID-19 as a pandemic.^1^ In the absence of proven therapies or a vaccine, public health departments, governments, employers, and healthcare institutions have taken measures to control the spread of the disease, including providing information and promoting nonpharmaceutical interventions, such as social distancing and hand washing. Given the novelty of the disease, information is rapidly evolving, with new evidence often contradicting earlier findings. These inconsistencies create uncertainty, leading to a need for trustworthy, health-related information.

### Information needs

Timely and accurate public health information related to COVID-19 is universally needed across stakeholders. Organizations have been asked to provide information on COVID-19, its symptoms, how it spreads, strategies for prevention, and how each organization is responding to the pandemic. Trusted sources of health information, like medical practices, have limited in-person visits to focus on treating the sick and reducing disease spread. Staff reductions have further compounded availability to answer questions. The pervasiveness of the pandemic has resulted in organizations assuming new roles related to the dissemination of public health information. Given the enormous demand for information about COVID-19, many stakeholders have leveraged emerging conversational technologies to automate responses to common COVID-19 related questions and information needs specific to their organizations.

### Chatbots in healthcare

One way to scale dissemination of COVID-19 related information is through technologies that employ natural language conversation. Chatbots, sometimes called conversational agents or virtual assistants, often differ in functionality. Consensus on a taxonomy of these conversational technologies is lacking.^2^^,^^3^ The simplest chatbots are capable of matching a predetermined set of topics with predefined answers, whereas more sophisticated conversational agents expand on their functionalities to employ machine learning and natural language processing (NLP) to understand questions in everyday language and engage users in increasingly complex conversations. For example, conversational agents may understand meaning, maintain context in dialogue, and learn with time to improve their performance. Some describe conversational agents that aid users in performing specific tasks as virtual assistants; they often have a characteristic personality expressed by tone, dialect, or style in conversation. Such conversational tools have demonstrated promise in clinical applications,^4^ including chatbots for determining social needs^5^ and panic disorder,^6^ as well as conversational agents for irritable bowel syndrome^7^ and behavior change.^8^ For the COVID-19 pandemic, conversational agents have been deployed to answer questions and to triage symptoms, but studies of their adoption and use to address questions surrounding COVID-19 have been limited to single institution experiences.^9–11^

### Study objective

We sought to characterize the diverse use cases of COVID-related conversational agents built using the Watson Assistant (WA) platform between March 30, 2020 and August 10, 2020. We measured the adoption through the number of users and messages sent. We determined the average number of conversational turns, with 1 turn representing 1 question–response pair.

## MATERIALS AND METHODS

### WA description and capabilities

WA is a platform for developing, training, and customizing conversational agents. Although not specific to the healthcare domain, this platform has previously been applied to medication prescribing, mental health, and Parkinson’s disease.^12–14^ Core natural language capabilities include: (1) understanding input, (2) classifying topics, (3) state management and maintaining a structured dialog (eg, functions to support dynamically collecting multiple pieces of information, digressions for allowing users to change topics without losing their place in the conversation, and disambiguation to clarify when users say something for which the system has multiple relevant responses^15^); and (4) retrieving information from a knowledge base through search. WA uses NLP and machine learning in the intent understanding, entity extraction, query expansion, and finding answers through estimating document relevancy. Search capacities also have NLP in its natural language understanding capabilities both when breaking down the user’s query as well as finding answers in documents. WA supports building a conversational interface into any application, device, or channel such as a website or interactive voice recognition system.

WA conversational agents allow users to initiate a conversation by entering questions. For example, when a user enters a question about COVID-19, a conversational agent built using WA will interpret the question to identify the *intent* (target of a user’s query) and match it to an internal list of intents and *entities* (for example, a condition) that answer the question within the dialogue interface or find the most relevant answer in its knowledge base.

WA is built upon a core set of functionalities with 3 main components that facilitate dialogue with users. The first component is the intent, which defines the type of information sought. The second component includes an entity that is used to provide a precise response for an intent. The final component is dialogue, which is the actual conversation a user has with the conversational agent. WA’s proprietary NLP capabilities facilitate creation and training of conversational agents with a minimal amount of data. Agents can be delivered in any cloud environment, allowing users to maintain ownership and privacy of their data. The technical details of functionalities and implementation are beyond the scope of this brief communication but are provided elsewhere.^16^

### Conversational agents for COVID-19

Beginning in March 2020, IBM offered a program called Citizen Assistant to any organization worldwide, including WA for COVID-19 and assistance with initial setup at no charge for at least 90 days, as part of IBM’s corporate social responsibility initiatives in response to the pandemic. WA is also free to use for anyone, for up to 10 000 messages per month and 1000 users per month. Conversational agents built using WA were trained to understand and respond, through both voice and text, to common COVID-19 questions, leveraging evidence-based sources where possible, such as guidance from the United States (US) Centers for Disease Control and Prevention (CDC). Basic COVID-19 content was made available in both English and Spanish.

WA provides both human-curated, predetermined responses as well as capabilities to dynamically search for and identify information from unstructured documents or websites on a scheduled basis. This architecture provides users with access to the most up-to-date information as science evolves and ensures some level of quality in the information provided through expert validation when needed. To dynamically search for and provide up-to-date information, WA treats the user input as a search query. It finds information that is relevant to the query from an external data source, such as the CDC, and returns it to the user.^17^

Conversational agents built using WA can be customized for specific use cases. For example, conversational agents can be trained to include information related to a specific language, locale, or organization, such as links to local school closings, local news, and state websites. Once the assistant is live and users ask questions, a human will typically review subsets of conversations for knowledge gaps. The assistant is retrained to answer any questions it was not initially trained on to cover these gaps.

### Conversational intents

An initial catalog of COVID-19 intents was created by experts in conversational agent design to cover areas including testing, case counts, travel restrictions, preventative behaviors, symptoms, and contact information. The content was based on current evidence and best practices retrieved from the CDC, Department of Labor, World Health Organization (WHO), and USA.gov. Intents were implemented as static responses or dynamic searches, depending on the types and sources of information, as well as how often this information changes. Relatively consistent, high priority COVID-19 knowledge intents were curated by humans, with intents and responses independently reviewed and evaluated by 2 physicians for face validity related to public health and clinical acceptability.

Disagreements were resolved through discussions with a third physician to reach consensus. Biweekly reviews to iteratively refine all intents and responses with clinicians and public health experts were also conducted and are ongoing. Intents with reliable sources of information and rapidly-changing answers (eg, case counts) were implemented with dynamic search or lookup functions, with data sources routinely reviewed by experts. Additional intents specific to organizational information needs and use cases were developed, such as intents covering physician and medical center access for providers, intents for testing coverage and premium payments for health plan members, and intents relating to when and how employees may work or return to work for employers (see Supplementary Table 1 file for a full characterization of intents). 

### Evaluation of usage

We assessed the initial success of the WA platform in delivering information for COVID-19 across use cases by measuring: (1) adoption of WA conversational agents through number and diversity of users, (2) the total number of messages (ie, number of times a conversational agent provides text to the user); and (3) the average number of conversational turns per session. These metrics were collected over a 4-month period between March 30, 2020 and August 10, 2020. Analysis was performed in RStudio version 3.6.1. For any organizations that joined the free trial after March 30, 2020, we calculated usage metrics from the date of initial use of WA for COVID-19.

## RESULTS

### Usage

All institutions achieved end-to-end deployment in approximately 3 weeks or less; the average time to initial use was 5 business days. Two implementations were voice-based, requiring users to call the implementing organization’s contact number, while the rest were web chat integrations. Each web-based agent was made available either through webchat on the organizations’ home sites or internal landing page (for employer organizations). The type of information provided ranged widely including: (a) COVID-19 symptoms, (b) testing information, (c) information on preventative behaviors, (d) local and national information about the disease, (e) response initiatives, (f) availability of services and how to access them, (g) guidelines, restrictions, closures, and reopening information, (h) course and exam information, (i) unemployment benefits and information, (j) stimulus payments, (k) business assistance, and (l) volunteer opportunities.

**Figure 1.**
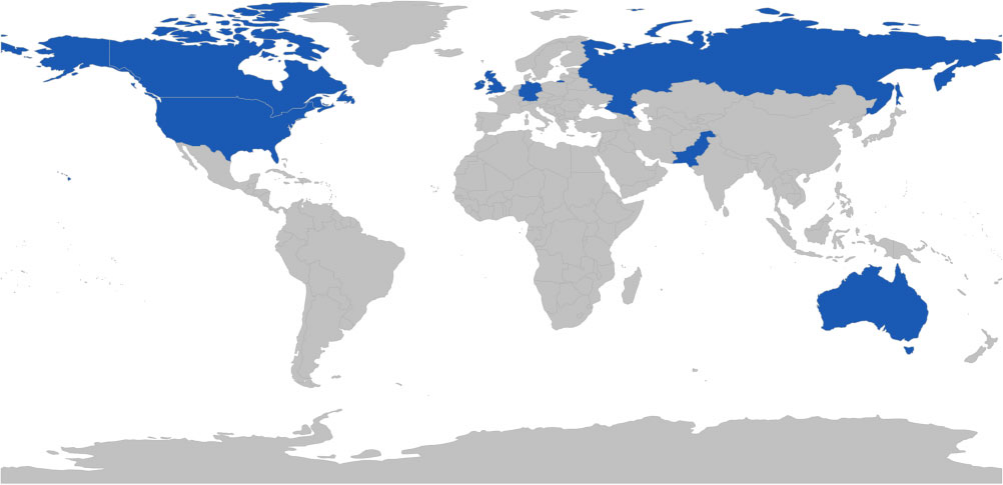
Countries with implementations of Watson Assistant for COVID-19 response.

As of 8/10/2020, 101 organizations had used WA for COVID-19 to develop their own conversational agent; of these, usage data for this study were available for 37 institutions in 9 countries. The types of organizations implementing conversational agents through the WA for COVID-19 platform were primarily governmental (N = 24), employers (N = 7), providers (N = 5), and health plans (N = 1). Most organizations leveraging this technology were located in the U.S. and Canada (N = 29), Europe (N = 4), and Asia Pacific (N = 4). Figure 1 shows countries where organizations implemented conversational agents using WA for COVID-19. The number of estimated potential users ranged from 26 000 to 212 000 people. The types of users included patients, health plan beneficiaries, students and staff, business owners and employees, and the general public (country, state, county, and city residents). Supplementary Table 2 further describes the organizations and their users.

**Figure 2.**
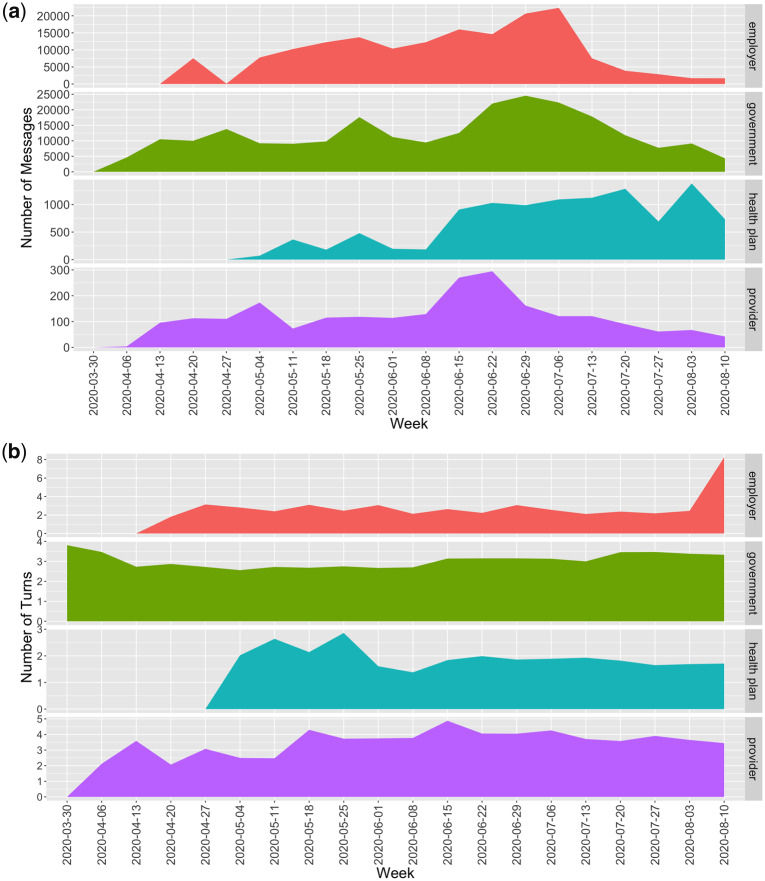
Usage metrics over time by organizational type: a) mean number of messages by week and b) mean number of conversational turns by week.

Total message usage and average number of conversational turns per session are presented in Table 1, with a visualization of these data over time for each organizational type in Figure 2. A total of 6 872 021 messages were sent in conversations about COVID-19 using the conversational platform. Mean conversational turns were highest for provider organizations (mean, 3.5 turns) and lowest for health plans (mean, 1.9 turns).

**Table 1.** Usage metrics from March 30, 2020 to August 10, 2020

Organization Type	Total Number of Messages	Mean Number of Messages*	Mean Conversational Turns*
Government (N = 24)	5 702 811	11 880 (min = 1; max = 24 557)	3.0 (min = 2.56; max = 3.8)
Employer (N = 7)	1 159 304	9742 (min = 109; max = 22 334)	2.9 (min = 1.8; max = 8.2)
Provider (N = 5)	11 379	120 (min = 4; max = 295)	3.5 (min = 2.1; max= 4.9)
Health Plan (N = 1)	10 710	714 (min = 71; max = 1 382)	1.9 (min = 1.4; max = 2.9)

* Calculated by week

## DISCUSSION

This brief communication describes rapid and widespread deployment, adoption, and usage of a set of conversational agents to address the overwhelming information needs created by COVID-19. We show that conversational agents built to answer many different types of questions for COVID-19 pandemic response can be deployed quickly and were broadly adopted during the early stages of the pandemic.

The COVID-19 pandemic generated an urgent need to provide answers to questions based on rapidly evolving scientific evidence. Citizens continue to want quick access to information in a manner that allows them to make informed decisions on how to protect themselves, their families, and their communities.^18^ To address these needs, several institutions have reported leveraging conversational agent technologies. Most of these agents focused on symptom self-checking for patient triage or mental health,^19^^,^^20^ while automating answers to common questions was more limited. This manuscript describes a platform used to deploy conversational agents to address a diverse set of information needs for a wide variety of stakeholders including governments, employers, providers, and health plans.

Thirty-seven organizations in 9 different countries implemented agents and delivered over 6.8 million messages, indicating widespread geographic adoption of these conversational agents and demand for public health information related to the COVID-19 pandemic. Published studies of conversational agents to address COVID-19 have previously been limited to single-institution experiences with a single conversational agent.^19^ Our experience demonstrates the ability of a conversational technology platform to support varied COVID-19 information needs across multiple institutions, representing diverse stakeholders and users.

Further, we report on conversational turns, which are used to assess the amount of interaction between a user and a system. The mean number of conversational turns per session was 2 to 3, indicating engagement with agents and suggesting they can answer most user questions efficiently. The relative number of turns may also underscore the complexity of some user questions, particularly clinical ones, since provider organizations had the most turns per session. Yet, across organizations, the number of conversational turns is not reflective of highly complex conversations. Due to the novel and rapidly evolving context in the early stages of a pandemic, most users probably asked simple, transactional types of questions such as “Is the hospital open?” and “What is COVID-19?” This trend is likely to change as the pandemic evolves. For example, in the later weeks of this study, conversational length among employers spiked (see Figure 2). We hypothesize that as workers returned to work, more complex conversations around workplace safety and reopening policies occurred.

### Limitations

This preliminary work has several limitations. This description of initial usage did not measure outcomes such as user satisfaction, frequency of intents, whether user questions were answered, or time and cost savings; these are topics of ongoing research. The manuscript reports the adoption and use of a system that is commercially available for enterprise solutions. However, this manuscript reported usage only during the period for which the platform was freely available as part of a philanthropic response to the pandemic, and the platform is freely available to anyone for low to medium volume applications.

## CONCLUSION

We have demonstrated the ability of a wide variety of organizations including governments, employers, providers, and payers to use conversational technologies to provide current information related to COVID-19 to their citizens, employees, patients, and beneficiaries. The WA platform enabled rapid implementation of a set of conversational agents for a wide variety of use cases, and usage data show demand for and adoption of these technologies during a rapidly evolving public health crisis. Our ongoing research aims to examine user conversations and how conversations change over time during the course of a pandemic. We are also investigating user satisfaction and experience with COVID-19 conversational agents.

## FUNDING

This study is funded by IBM Watson Health.

## AUTHOR CONTRIBUTIONS

MMc contributed to the conception of the work; the acquisition, analysis, and interpretation of data; the drafting of the work; and critical revision. BS and AP contributed to the interpretation of data, the drafting of the work, and critical revision. MMa contributed to the acquisition and interpretation of data; the drafting of the work; and critical revision. GP contributed to the conception of the work, drafting of the manuscript, and critical revision.

## Supplementary Material

ocaa316_Supplementary_Data
