# Supplementary material for: Leveraging conversational technology to answer common COVID-19 questions
Source: J Am Med Inform Assoc. 2021 Feb 1;28(4):850–5. doi: 10.1093/jamia/ocaa316 (PMC7798957; doi:10.1093/jamia/ocaa316)
Supplement: ocaa316_Supplementary_Data [file ocaa316_supplementary_data.pdf]

Supplement to:

“Leveraging Conversational Technology to Answer Common COVID-19 Questions”

Contents:

This supplement contains a list and descriptions of the pre-trained intents that are immediately available to users of Watson Assistant for COVID-19. It also contains a table to summarize the types of users and use cases of the Watson Assistant for COVID-19 platform.

Table of Contents

Table 1. WA for COVID-19 Standard Intents...page 2
Table 2. Description of Use Cases...page 9

Table 1. WA for COVID-19 Standard Intents.

Intent Name	Intent Description	Sample User Input Questions	Response	Organization Type	Validated by Medical Experts
COVID Symptoms	Informs user as to the symptoms	What are symptoms ?	If you think you have been exposed to COVID-19 and develop a fever and symptoms such as cough or difficulty breathing, call your healthcare provider for medical advice. According to the CDC, reported illnesses have ranged from mild symptoms to severe illness and death for confirmed coronavirus disease 2019 (COVID-19) cases. The most common symptoms of COVID-19 are fever, dry cough, shortness of breath, chills, muscle pain, headaches, sore throat or new loss of smell or taste. These symptoms may appear 2-14 days after exposure. If you develop emergency warning signs for COVID-19 get medical attention immediately. Emergency warning signs include: difficulty breathing or shortness of breath, persistent pain or pressure in the chest, new confusion or inability to arouse, bluish lips or face. This list is not all possible symptoms. Other less common symptoms have been reported, including gastrointestinal symptoms like nausea, vomiting, or diarrhea. Please consult your medical provider for any other symptoms that are severe or concerning. For more information, visit your state health department: <link> or CDC website: <link>.	All	Yes
Testing Triage	Informs user whether they need to get tested	Do I need to get tested?	Not everyone needs to be tested for COVID-19. Here is some information that might help you make decisions about seeking medical care or testing. Most people have mild illness and are able to recover at home without medical care. They may not need to be tested. CDC has guidance for who should be tested, but decisions about testing are at the discretion of state and local health departments and/or individual clinicians.		

Intent Name	Intent Description	Sample User Input Questions	Response	Organization Type	Validated by Medical Experts
Testing Locations	Directs user to testing location and information on how to get tested	Where can I get tested? How do I get tested?	If you are a close contact of someone with COVID-19 or you are a resident in a community where there is ongoing spread of COVID-19 and develop symptoms of COVID-19, call your healthcare provider and tell them about your symptoms and your exposure. They will decide whether you need to be tested, but keep in mind that there is no treatment for COVID-19 and people who are mildly ill may be able to isolate and care for themselves at home. For more information, visit your state health department or CDC website: <link>. The process and locations for testing vary from place to place. Contact your state, local, tribal, or territorial department for more information, or reach out to a medical provider. You can find a list of State and Local Health Departments by clicking on the provided CDC: <link>	All	Yes
Testing Results	Informs where and when individuals will receive results from their taken test	Where do I find out my test results for COVID-19?	For the state lab testing center, individuals will be contacted directly with their results within 72 hours, depending on test volume. Do not call the state lab, the state health department, or a local public health agency to get your results. Otherwise, the person who requests the tests will receive the results. If your doctor submitted your test to the lab, your doctor will receive your results and communicate those results to you. For more information, go to: <link>	All	Yes
Case Counts	Informs the number of cases within a location	Where are cases? Are there cases of corona virus near me?	Please monitor your state's Department of Health website to view the latest list of COVID-19 cases. You can view the list of COVID-19 cases in the US by clicking on the following: <link>.<br>You can view the number of cases anywhere in the world on the by clicking on the following: <link>	All	Yes

**Intent Name**	**Intent Description**	**Sample User Input Questions**	**Response**	**Organization Type**	**Validated by Medical Experts**
Travel Restrictions	Provides information on travel	Can I travel?	For information go to: <link>	All	Yes
Cleaning and Disinfecting	Instructs users how to clean to prevent spreading infection	How do I clean my home properly?	To disinfect, most common EPA-registered household disinfectants will work. Use disinfectants appropriate for the surface. Options include diluting your household bleach. To make a bleach solution, mix: 5 tablespoons (1/3rd cup) bleach per gallon of water OR 4 teaspoons bleach per quart of water Follow manufacturer's instructions for application and proper ventilation. Check to ensure the product is not past its expiration date. Never mix household bleach with ammonia or any other cleanser. Unexpired household bleach will be effective against coronaviruses when properly diluted. Products with EPA-approved emerging viral pathogens are expected to be effective against COVID-19 based on data for harder to kill viruses. Follow the manufacturer's instructions for all cleaning and disinfection products (e.g., concentration, application method and contact time, etc.). Visit the CDCs website for further instructions on cleaning and disinfecting. The CDC provides great information on how to clean and disinfect your home. They are always updating their information, so you should check out: <link>	All	Yes

Intent Name	Intent Description	Sample User Input Questions	Response	Organization Type	Validated by Medical Experts
Protecting Against Infection	Provides guidance on precautionary measures to protect against virus.	How do I protect myself?	Wash your hands often with soap and water for at least 20 seconds especially after you have been in a public place, or after blowing your nose, coughing, or sneezing. If soap and water are not readily available, use a hand sanitizer that contains at least 60% alcohol. Cover all surfaces of your hands and rub them together until they feel dry. Avoid touching your eyes, nose, and mouth with unwashed hands. Avoid close contact with people who are sick. The best way to avoid getting sick is to limit your exposure to others. COVID-19 is thought to spread mainly through person to person transmission, so it's important to distance yourself from others as much as possible to prevent getting the virus. Additionally, you should wash your hands often and avoid touching your face as much as possible. For more information, check out the CDC's guide to protecting yourself and others: <link>. You could spread COVID-19 to others even if you do not feel sick. Everyone should wear a cloth face cover when they have to go out in public, for example to the grocery store or to pick up other necessities. Cloth face coverings should not be placed on young children under age 2, anyone who has trouble breathing, or is unconscious, incapacitated or otherwise unable to remove the mask without assistance. The cloth face cover is meant to protect other people in case you are infected. Do NOT use a facemask meant for a healthcare worker. Continue to keep about 6 feet between yourself and others. The cloth face cover is not a substitute for social distancing.	All	Yes
Connect To Agent	Direct user to a human agent	I want a person	Okay, let me get you connected to one of our team members who can assist you further.	All	No

Intent Name	Intent Description	Sample User Input Questions	Response	Organization Type	Validated by Medical Experts
Prevent COVID Spread	Describe steps to take to lessen the spread of the virus	What do I do to keep the coronavirus from spreading?	The virus spreads mainly between people who are in close contact with one another (within about 6 feet) through respiratory droplets produced when an infected person coughs or sneezes. It also may be possible that a person can get COVID-19 by touching a surface or object that has the virus on it and then touching their own mouth, nose, or possibly their eyes, but this is not thought to be the main way the virus spreads. For additional information, go to: <link>. For information on how to protect your family and friends if you are contagious go to <link>.	All	Yes
Test Center Contact Info	Locate a physical test center or an address.	Where is your test center?	The process and locations for testing vary from place to place. Contact your state, local, tribal, or territorial department. For more information: <link> or reach out to a medical provider. State and local public health departments have received tests from CDC while medical providers are getting tests developed by commercial manufacturers. While supplies of these tests are increasing, it may still be difficult to find someplace to get tested. For more information, go to: <link>	All	Yes
COVID Comparing Illnesses	Compare illnesses like Influenza, SARS, MERS, to COVID-19	"What's the difference between flu and coronavirus?"	The World Health Organization's director-general gave a great overview of the differences between COVID-19 and many commonly known illnesses, you can read all kinds of interesting details in the summary: <link>. For additional information about similarities and differences between Flu and COVID-19, visit: <link>.	All	Yes

Intent Name	Intent Description	Sample User Input Questions	Response	Organization Type	Validated by Medical Experts
COVID Shortness of Breath Definition	Define shortness of breath (since this is a COVID symptom)	"How do I know what shortness of breath is"	Shortness of breath or difficult breathing is a feeling of uncomfortable breathing or like you are not getting enough air. <link> Emergency warning signs include: trouble breathing, persistent pain or pressure in the chest, new confusion or inability to arouse, bluish lips or face. * This list is not all inclusive. If you develop emergency warning signs for COVID-19 get medical attention immediately. For more information visit: <link>	All	Yes
No Doctor	Help those who do not have primary care physicians	Who do I call if don't have a PCP?<br>Who should I call if I'm sick?	If you do not have health insurance or lose coverage, check your state health department for information about walk-in / convenient care clinics or urgent care centers. If you can't afford healthcare cost for these centers, please contact local community health centers, rural health clinics, and safety net health centers. For more information on state health departments, go to: <link>. Virtual Care is also a great option for care. It enables patients to seek medical attention from their home while limiting the spread of germs. You can learn more about the Virtual Care service from <link>.	Provider	Yes
Hospital Visitation	Provides guidance on current visitation policy.	Can I visit family members in the hospital?<br>Have you restricted patient visits?	Unfortunately, we have had to create a restricted visitor policy that we are updating each day <link>	Provider	No

Intent Name	Intent Description	Sample User Input Questions	Response	Organization Type	Validated by Medical Experts
Member Testing Eligibility	Provide guidance on eligibility for COVID-19 testing	Is this test covered? What is the cost of the test?	Your copayments, coinsurance and deductibles will be waived for the test to diagnose COVID-19. COVID-19 testing typically requires an order from a healthcare professional. A healthcare provider can determine if testing is appropriate based on your risks and symptoms. Your healthcare provider does NOT need to call for authorization to order a test that they deem necessary.	Health Plan/Member	Yes
Covid Member Billing	Educate members concerned about paying their monthly premium	Can I skip my premium payment this month?	Determined by the organization	Health Plan/Member	No
Worker Options	Educate employees when testing is instated*	If I test positive, how long will I be out of work?	If you test positive for COVID-19, please work with your healthcare provider for the proper care plan based on the CDC Guidelines: <link>. Virtual Care is also a great option for care. It enables patients to seek medical attention from their home while limiting the spread of germs. You can learn more about the Virtual Care service. Your employer also has a number of options available to you during this time including assigning a case manager to help coordinate your care. If you are a health care provider with suspected or confirmed COVID-19, go to <link>.	Employer	Yes

* Educate employees during back-to-work initiatives when testing is instated prior to returning to work.

Table 2. Description of Use Cases.

Organization Type	Country	Implementation Type	Number of Estimated Potential Users*	Information Type	User Type
Digital Strategy and Consulting Services	Australia	web	2,300,000	• Symptoms of COVID-19<br>• How to get tested<br>• School closures<br>• Guidance on events and large Gatherings	Clients
University	Australia	web	36,000	• COVID-19 information<br>• Access to recorded lectures<br>• Exam information<br>• Student wellbeing services	Students and staff
Provider	Canada	web	15,000,000	• Travel restrictions<br>• Symptoms<br>• Testing<br>• Changes to services<br>• Reopening framework	Patients

Organization Type	Country	Implementation Type	Number of Estimated Potential Users*	Information Type	User Type
City Government	Canada	web	340,000	• Symptoms of COVID-19<br>• How to get tested<br>• School closures<br>• Guidance on events and large gatherings	City residents
University	Canada	web	30,000	• Symptoms of COVID-19<br>• Financial support<br>• Reopening information<br>• School applications<br>• Learn about programs	Students and staff
Government	Germany	web	72,000,000	• Symptoms of COVID-19<br>• How to get tested<br>• Guidelines and restrictions	Country residents and citizens

Organization Type	Country	Implementation Type	Number of Estimated Potential Users*	Information Type	User Type
County Government	Ireland	web	300,000	• Community Response Initiative<br>• Availability of services through the COVID-19 crisis	County residents
Federal Government Agency	Pakistan	web	212,000,000	• Symptoms of COVID-19<br>• How to Get Tested<br>• Guidelines and restrictions	Country residents and citizens
University	Russia	web	50,000	• COVID-19 information<br>• Course information<br>• Exam information	Students and staff
Industry Association	Singapore	web	26,000	• Symptoms of COVID-19<br>• How to get tested<br>• Guidelines and restrictions	Business owners

Organization Type	Country	Implementation Type	Number of Estimated Potential Users*	Information Type	User Type
Consulting, Digital and Software Solutions	United Kingdom	web	63,000	• Symptoms of COVID-19<br>• How to get tested<br>• Guidelines and restrictions	Clients
State Government Department of Labor	USA	web	13,000,000	• Unemployment benefits information<br>• COVID-19 information	State residents
State Government	USA	web	11,000,000	• Symptoms of COVID-19<br>• How to get tested<br>• School closures	State residents
State Government	USA	web	10,000,000	• Face coverings<br>• Gatherings, events & capacity limits<br>• Quarantine & isolation<br>• Travel restrictions<br>• Unemployment	State residents

Organization Type	Country	Implementation Type	Number of Estimated Potential Users*	Information Type	User Type
State Innovation Office	USA	web	9,000,000	• COVID-19 symptoms<br>• Business assistance<br>• Testing sites	State business owners and residents
State Government	USA	web	6,000,000	• Governor's Executive Order<br>• COVID-19 data<br>• How to get tested<br>• COVID-19 symptoms<br>• Unemployment help	State residents
State Government	USA	web	5,000,000	• Travel restrictions<br>• Symptoms<br>• Testing	State residents
State Government	USA	web	4,000,000	• Symptoms<br>• How to get tested<br>• School closures<br>• Guidance on events<br>• Business rules<br>• Travel restrictions	State residents

Organization Type	Country	Implementation Type	Number of Estimated Potential Users*	Information Type	User Type
City Government	USA	voice	3,000,000	• Symptoms of COVID-19<br>• How to get tested	City residents
State Government	USA	web	2,000,000	• Travel restrictions<br>• Symptoms<br>• Testing	State residents
State Secretary	USA	web	2,000,000	• COVID-19 information<br>• Voting information	State residents
City Government	USA	web	1,000,000	• CDC information<br>• Local news updates<br>• Local screening<br>• Business closures	City residents
Health Plan	USA	web	1,000,000	• Symptoms of COVID-19<br>• How to get tested<br>• Recent news<br>• Travel restrictions<br>• Symptoms<br>• Testing	Beneficiaries

Organization Type	Country	Implementation Type	Number of Estimated Potential Users*	Information Type	User Type
State Government Labor Department	USA	voice	730,000	• Stimulus payments<br>• Unemployment application and benefits	State residents
County Government	USA	web	600,000	• Testing sites<br>• Test results<br>• Policies and restrictions	County residents
City Government	USA	web	500,000	• Travel restrictions<br>• Symptoms<br>• Testing	City residents
Shipping company	USA	web	400,000	• COVID-19 Information<br>• Business policies to address the pandemic	Employees
Health Technology Company	USA	web	350,000	• COVID-19 Information<br>• Business policies to address the pandemic	Employees

Organization Type	Country	Implementation Type	Number of Estimated Potential Users*	Information Type	User Type
Provider	USA	web	250,000	• Testing sites<br>• Test results<br>• Policies and restrictions	Patients
City Government	USA	web	200,000	• Travel restrictions<br>• Symptoms<br>• Testing	City residents
Manufacturing Company	USA	web	190,000	• COVID-19 Information<br>• Business policies to address the pandemic	Employees
County Government	USA	web	180,000	• COVID-19 initiatives<br>• Testing sites<br>• Volunteering<br>• Business closures<br>• Symptoms	Country residents
County Government	USA	web	140,000	• Travel restrictions<br>• Symptoms<br>• Testing	County residents

Organization Type	Country	Implementation Type	Number of Estimated Potential Users*	Information Type	User Type
County Government	USA	web	100,000	• Travel restrictions<br>• Symptoms<br>• Testing	County residents
Provider	USA	web	74,000	• Symptoms<br>• How to get tested<br>• Treatment information<br>• Travel restrictions	Patients
Provider	USA	web	70,000	• Travel restrictions<br>• Symptoms<br>• Testing<br>• Changes to services<br>• Reopening framework	Patients
Provider	USA	web	37,000	• Symptoms<br>• How to get tested<br>• Telehealth information<br>• Patient resources<br>• Finding a doctor	Patients

* Upper bound of the number of individuals or clients which an organization serves
